# The Individuals’ Willingness to Get the Vaccine for COVID-19 during the Third Wave: A Study on Trust in Mainstream Information Sources, Attitudes and Framing Effect

**DOI:** 10.3390/bs12100399

**Published:** 2022-10-18

**Authors:** Marianna Masiero, Davide Mazzoni, Silvia Francesca Maria Pizzoli, Simone Gargenti, Roberto Grasso, Ketti Mazzocco, Gabriella Pravettoni

**Affiliations:** 1 Applied Research Division for Cognitive and Psychological Science, IEO European Institute of Oncology, IRCCS, 20141 Milan, Italy; 2 Department of Oncology and Hemato-Oncology, University of Milan, 20122 Milan, Italy; 3 MSc Scienze Cognitive e Processi Decisionali, University of Milan, 20122 Milan, Italy

**Keywords:** decision-making, framing effect, vaccine intention, COVID-19, attitudes, trust

## Abstract

Different inner and external determinants might explain an individual’s willingness to get the vaccine for COVID-19. The current study aims at evaluating the effects of trust in mainstream information sources on individuals’ willingness to get the vaccine and the moderator role of the message framing. Six hundred and thirty-four participants (68.5% females and 31.5% males) were enrolled in an online survey. Participants filled out a questionnaire assessing: trust in mainstream information sources and vaccinal attitude (trust in vaccine benefit, worries over unforeseen future effects, concerns about commercial profiteering, and preference for natural immunity). In addition, participants were randomly exposed to one of four conditions of framing information about the vaccine (gain-probability; gain-frequency; loss-probability; loss-frequency). Results showed that trust in vaccine benefit (b = 9.90; 95% CI: 8.97, 11.73) and concerns about commercial profiteering (b = −4.70; 95% CI: −6.58, −2.81) had a significant effect on the intention to get the vaccine. Further, a significant interaction was observed between loss-gain and trust in vaccine benefit and between frequency-probability and concerns about commercial profiteering. Future vaccination campaigns should consider the individuals’ concerns about vaccine benefit and economic profits to efficaciously deliver frequency-framed or probability-framed information.

## 1. Introduction

The pandemic of severe acute respiratory disease SARS-CoV-2 (also named COVID-19), which emerged in late December 2019 in China, still affects the entire world with dramatic health and economic costs, with more than six million deaths. Worldwide efforts have been made to try to control the spreading of the virus, and numerous vaccine development programs (using different types of vaccines based on mRNA or viral vectors) have started in response to this emergency [1]. As vaccines became available between 2019 and 2020, individual protective choices became crucial, and many governments promoted vaccination campaigns, delivering scientific information with the support of experts and the media [2,3]. However, in many countries, a significant part of the population keeps a negative attitude toward vaccines and would not accept receiving a vaccine [4,5,6,7]. To explain the hesitancy in getting vaccinated against COVID-19, several factors should be considered, both related to the individual’s beliefs and the characteristics of the pro-vaccination messages.

First, individuals’ trust in key informational sources such as the national governments and health authorities is a critical determinant of intention to be vaccinated [8]. Indeed, as reported in previous studies, higher trust in health authorities is a key predictor of vaccine acceptance, contributing to better adherence to recommended actions [9]. This is consistent with the extensive social psychological literature, which largely investigated the role of source credibility, especially in health communication [10,11], suggesting that trust in the pro-vaccine informational sources may promote more positive attitudes toward the vaccine [12].

In this sense, another group of factors to consider is the individuals’ attitudes toward COVID-19 [2]. Vaccinal attitude can be well described as a multidimensional construct rather than unidimensional, as individuals who refuse to get the vaccines may have worries about unusual medical interventions, the safety of vaccines, or mistrust of pharmaceutical corporations [13]. In this regard, Leslie and Petrie (2017) proposed a model of vaccinal attitude that is based on four distinct but correlated dimensions: (1) mistrust of vaccine benefit, (2) worries about unforeseen future effects, (3) concerns about commercial profiteering, and (4) preference for natural immunity. These factors were significantly related to prior vaccination behavior and future intentions to obtain recommended vaccinations [13].

Not only the individual’s trust in the information sources, which can be shaped by whether the information is related to the public or private sector or given by single individuals or group of experts [14] and the attitudes toward vaccination but also the framing used to display risks and benefits associated with the vaccine is a key determinant of the vaccine decisions [15]. As prior research in decision-making has highlighted, how the information is presented might influence individual health choices and vaccine behaviors. In detail, the specific framing used to describe treatment options might shape the construction of individual health preferences and, subsequently, the modulation of current and future health behaviors (Framing Effect) [16]. The effect of the framing of the information is significant when uncertainty levels are higher, and the information available is scarce, as in the context of the evolving COVID-19 pandemic.

For example, people typically prefer risk-averse choices when information is presented with a gain frame, while risk-seeking choices are preferred in the presence of a loss condition [17]. *Gain and loss framing* taps into emotional responses to information [17]. Indeed, a gain-framed message emphasizes benefits, while a loss-framed message the costs. Generally, in the case of illness detection behaviors, using a loss-framed communication is more effective (people are risk-averse). In contrast, in the case of illness prevention behaviors, gain-framed communication is more convincing (people are risk-seeking) [18,19]. Notwithstanding, the vaccine is considered a higher-hazard activity that might have serious health consequences (e.g., cerebral thrombosis and stroke) and is considered both a preventive and high-risk behavior. Few studies have applied the gain-loss framing on health-related COVID-19 pandemic messages to boost adherence to protective behaviors, and vaccine campaigns report conflicting results [20,21,22].

Besides gain and loss framing, the format of the presentation of choices and risks can shape individual behaviors. In this regard, the risks and benefits of medical decisions can be represented in *percentages or frequencies*, which, per se, might influence the interpretation by a person [23]. People react differently if the same phenomenon is described in the form of percentages or the form of frequencies [24,25]: percentages seem to be easier than comparing frequencies [26], while conditional probabilities are easier to be understood in the form of frequencies [24].

Commonly, studies on the intention to get vaccinations for other diseases have found mixed results when testing the impact of game-loss framing and frequency percentage. Some of them found an effect of the frame on the intentions to obtain the MMR vaccine (measles, mumps, and rubella) [27], HPV vaccination [28], and flu vaccine [29,30]. However, most of these studies found that such effects were mediated or moderated by other individual factors, suggesting that the impact of framing may also depend on individuals’ characteristics. Overall, understanding the effect of the framing on vaccine decisions requires additional validation [31].

With these premises in mind, the current study aimed to evaluate the effects of trust in the mainstream (pro-vaccine) information sources and attitudes on the individual’s willingness to get the vaccine in laypeople during the third wave of COVID-19. More specifically, we hypothesized that the psycho-cognitive attitudes of higher trust in mainstream information sources and more positive attitudes toward the vaccine would be positively associated with behaving in a preventive way, as measured by the individuals’ intention to get the vaccine.

Second, we hypothesized that this effect of trust in information sources on the intention to get the vaccine was mediated by the attitudes toward vaccination. In other words, higher trust in information sources would have a positive effect on the individual’s attitudes toward the vaccine, which would positively affect the intention to get the vaccine.

**Figure 1:**
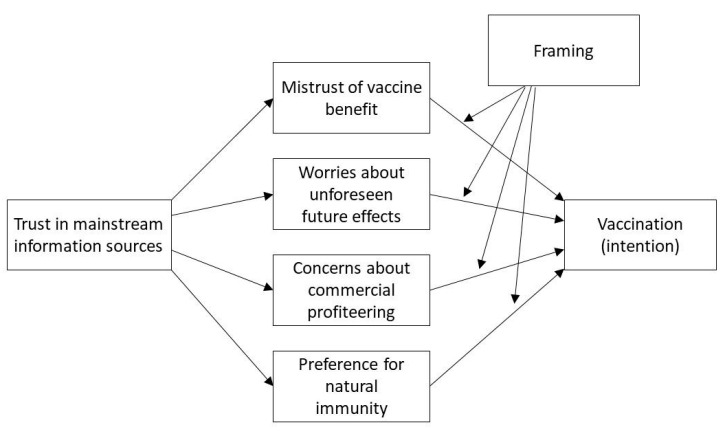
The hypothesized model.

Furthermore, a third exploratory aim was to assess the impact of message frames (gain vs. loss; frequency vs. percentage) on the willingness to get the vaccine. We thus hypothesized that a different frame (gain vs. loss, frequency vs. percentage) in the presentation of the COVID-19 vaccine could impact the individuals’ willingness to get the vaccine. Moreover, we wanted to test if a different frame could amplify or reduce the impact of different individual attitudes, that is, their possible moderator role (gain vs. loss, frequency vs. percentage). The hypothesized model is summarized and presented in Figure 1.

## 2. Materials and Methods

### 2.1. Procedure

Six hundred and thirty-four participants (68.5% female and 31.5% male) with a mean age of 22.59 (SD = 16.12) were enrolled in an online survey. At the time of data collection, most respondents (472; 74.4%) reported that they did not have personal experience with COVID-19, but 592 (93.3%) had at least some relatives (219; 34.5%) or friends (373; 58.8%) who did. The online questionnaires were collected via the Qualtrics platform through a network sampling method. The study employed a cross-sectional and prospective design. The link to the survey was published on social media at the beginning of Italy’s Plan for anti-SARS-CoV-2/COVID-19 vaccination (from January 2021 to April 2021). Participants were also invited to share the link with their acquaintances. Participants had to sign an online informed consent and then were invited to complete several questionnaires.

In detail, participants were asked to complete a questionnaire on their sociodemographic features and health status. In addition, each participant was required to complete a questionnaire assessing the following psychological constructs: trust in mainstream information sources, vaccinal attitude (trust in vaccine benefit, worries over unforeseen future effects, concerns about commercial profiteering, and preference for natural immunity). At the end of this first phase, participants were randomly assigned to one of four conditions of framing (respectively: Condition1 gain-probability; Condition2 Gain-Frequency; Condition3 loss-probability; Condition4 loss-frequency; Table 1), and then the willingness to get a vaccine has been evaluated using a visual analog scale. The time duration of the entire survey was 25 min.

### 2.2. Instruments

In the first part of the questionnaire, some background information was assessed: education, number of inhabitants in the living town, and yearly income. Perceived health status was assessed with a single item adapted from previous studies [32]: “How would you rate your current health status?”. The item had five possible response options ranging from 1 (very bad) to 5 (perfect).

The perceived *trust in information sources* was assessed through an ad-hoc scale, requesting the participants the amount of trust they have in four pro-vaccines information sources: the government, the research, the mass media, and the experts. Possible answers ranged from 0 (not at all) to 100 (complete). The reliability of the scale was acceptable (α = 0.72), and a mean index was thus used in the analyses.

*Vaccinal attitudes* were measured with a 12-item, four-factor scale by Martin and Petrie [13]. The subscales of the questionnaire are: trust in vaccine benefit (e.g., “I feel protected after getting vaccinated”) (α = 0.92); worries over unforeseen future effects (e.g., “Vaccines can cause unforeseen problems in children”) (α = 0.89); concerns about commercial profiteering (e.g., “Authorities promote vaccination for financial gain, not for people’s health”) (α = 0.93); preference for natural immunity (e.g., “Natural exposure to viruses and germs gives the safest protection”) (α = 0.86). Overall, the four subscales explain variances in concerns about vaccines and perceived utility.

The *framing effect* has been investigated widely in research using Kahneman and Tversky’s (1981) hypothetical scenario about contagious Asian disease [33]. Coherently, two dimensions were adopted to design four ad-hoc framed scenarios to evaluate the framing effect. The gain-framed scenarios were positive and highlighted the number of potential lives saved, while the loss-framed scenarios were negative and highlighted the number of potential lives lost. All the information provided was based on IHME (Institute of Health Metrics and Evaluation) projections about COVID-19- related deaths. Each participant was randomly assigned to read a message presented in a condition that was the combination of either a loss or gain and a frequency or probability frame (see Table 1).

*Intention to get vaccinated against COVID-19*. A visual analog scale (VAS) has been used to assess the intention to get the vaccine. The possible answers ranged from 0 (I will not get the vaccine for COVID-19) to 100 (I will get the vaccine for COVID-19).

### 2.3. Statistical Analyses

The analytical process followed different steps. First, descriptive statistics were computed for all the sociodemographic variables and the questionnaires’ scores.

Second, Pearson’s correlation and *t*-test were applied to assess the relationship between the key variables. Third, our main hypotheses were tested through a moderated mediation model with the Macro PROCESS 3.2, using 95% CI and 5.000 Bootstrap samples [34]. The intention to get vaccinated against COVID-19 was inserted as the dependent variable. Age, health status, and education were inserted as covariates. Trust in mainstream information sources was inserted as the main predictor, while the four dimensions of attitudes were inserted as mediators of the relationship between trust in mainstream information sources and intention. Finally, the two dichotomous variables of framing, loss-gain, and frequency-probability, were inserted as moderators of the relationship between attitudes and the intention to get the vaccination. All the data analyses were conducted through SPSS version 26.0.

## 3. Results

Table 2 presents means, standard deviations, and the correlations between the key variables under study. All the variables showed acceptable values of skewness (ranging from −2.29 to 1.21) and kurtosis (ranging from −0.60 to 4.53). All the correlations were significant in the expected direction, and both trust and the four dimensions of attitudes showed at least a weak correlation to getting vaccinated.

In terms of education, 178 participants (28.1%) had up to a high school degree, while 456 (71.9%) had an academic degree. The number of inhabitants in the town of residence was up to 10,000 for 219 participants (34.5%), between 10,001 and one million for 321 participants (50.6%), and more than one million for 94 participants (14.8%). The income (in thousands of euros) was below 15 for 172 (27.1%) participants, between 15 and 26 for 178 (28.1%) participants, and higher than 26 for 184 participants (44.8%). These three sociodemographic variables (education, number of inhabitants in the town of residence, and income) were considered in regard to the intention to get vaccinated. The group of participants with an academic degree showed a higher intention to get vaccinated (M = 91.24; SD = 28.22), compared to the participants with up to a high school degree (M = 81.03; SD = 28.22) (t = −4.40; *p* < 0.001). The number of citizens in the town of residence (F = 2.45; *p* = 0.087), as well as the income (F = 1.98; *p* = 0.14), were not associated with a different intention to get vaccinated.

The intention to get vaccinated was also considered in relation to the two framing dimensions (loss-gain and frequency-probability). The results of the two series of independent samples *t*-test did not report significant differences (*p* > 0.05). The possible interaction between the two framing conditions was also tested through ANOVA. However, neither the main effects of the two framing variables nor their interaction were significant (*p* > 0.05; data not shown).

### 3.1. Trust in Information Sources and Attitudes toward Vaccines

In the third step of the analysis, we tested the hypothesized model (see Figure 1) (R2 = 0.48; F = 24.90, *p* < 0.001). Regarding the left part of the model, the effect of trust in mainstream information sources was significant for all four dimensions of attitudes. More specifically, on trust in vaccine benefit (b = 0.03; 95% CI: 0.02, 0.03), worries about unforeseen future effects (b = −0.01; 95% CI: −0.02, −0.01), concerns about commercial profiteering (b = −0.03; 95% CI: −0.03, −0.02), preference for natural immunity (b = −0.02; 95% CI: −0.03, −0.02). Age, health status and education were also regressed on the four dimensions of attitudes. Age showed a significant effect on worries about unforeseen future effects (b = 0.01; 95% CI: 0.00, 0.02), but not on trust in vaccine benefit (b = 0.00; 95% CI: −0.00, 0.01), concerns about commercial profiteering (b = 0.00; 95% CI: −0.00, 0.01), and preference for natural immunity (b = 0.00; 95% CI: −0.00, 0.01). Health status showed a significant effect on trust in vaccine benefit (b = 0.19; 95% CI: 0.09, 0.28), concerns about commercial profiteering (b = −0.18; 95% CI: −0.29, −0.08), but not significant on worries about unforeseen future effects (b = −0.03; 95% CI: −0.15, 0.10) and preference for natural immunity (b = −0.08; 95% CI: −0.19, 0.03). Education showed a significant effect on all the four dimensions of attitudes: on trust in vaccine benefit (b = 0.19; 95% CI: 0.03, 0.34), worries about unforeseen future effects (b = −0.33; 95% CI: −0.54, −0.13), concerns about commercial profiteering (b = −0.32; 95% CI: −0.49, −0.15), preference for natural immunity (b = −0.30; 95% CI: −0.48, −0.12).

### 3.2. The Predictors of the Intention to Get Vaccinated

In terms of the right part of the same model, age and health status showed a non-significant effect on intention (age: b = 0.01; 95% CI: −0.09, 0.10; health status: b = −0.10; 95% CI: −20.04, 1.83). Education showed a significant and positive effect on intention (b = 4.22; 95% CI: 1.12, 7.32). Trust in mainstream sources of information showed a non-significant effect on intention (b = 0.06; 95% CI: −0.04, 0.15).

In terms of the four dimensions of attitudes, only trust in vaccine benefit (b = 9.90; 95% CI: 8.97, 11.73) and concerns about commercial profiteering (b = −4.70; 95% CI: −6.58, −2.81) showed a significant effect on intention, while worries about unforeseen future effects (b = −0.62; 95% CI: −1.97, 0.72) and preference for natural immunity (b = −1.14; 95% CI: −2.84, 0.56) did not.

**Figure 2:**
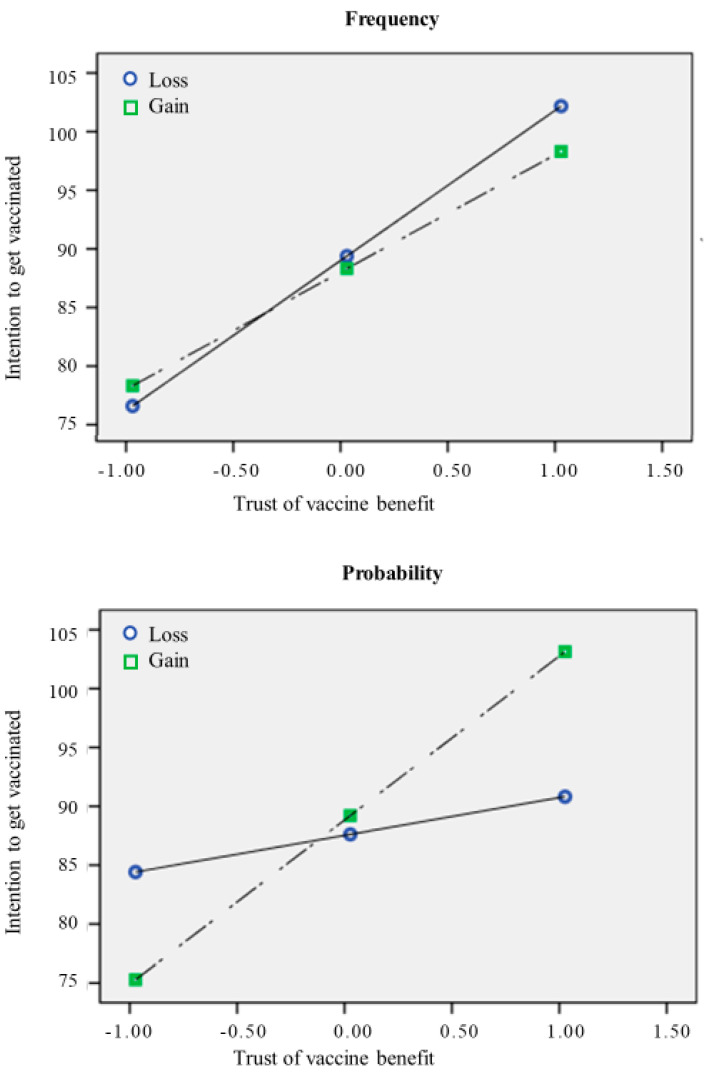
The relationship between trust in vaccine benefit and intention to get the vaccine.

**Figure 3:**
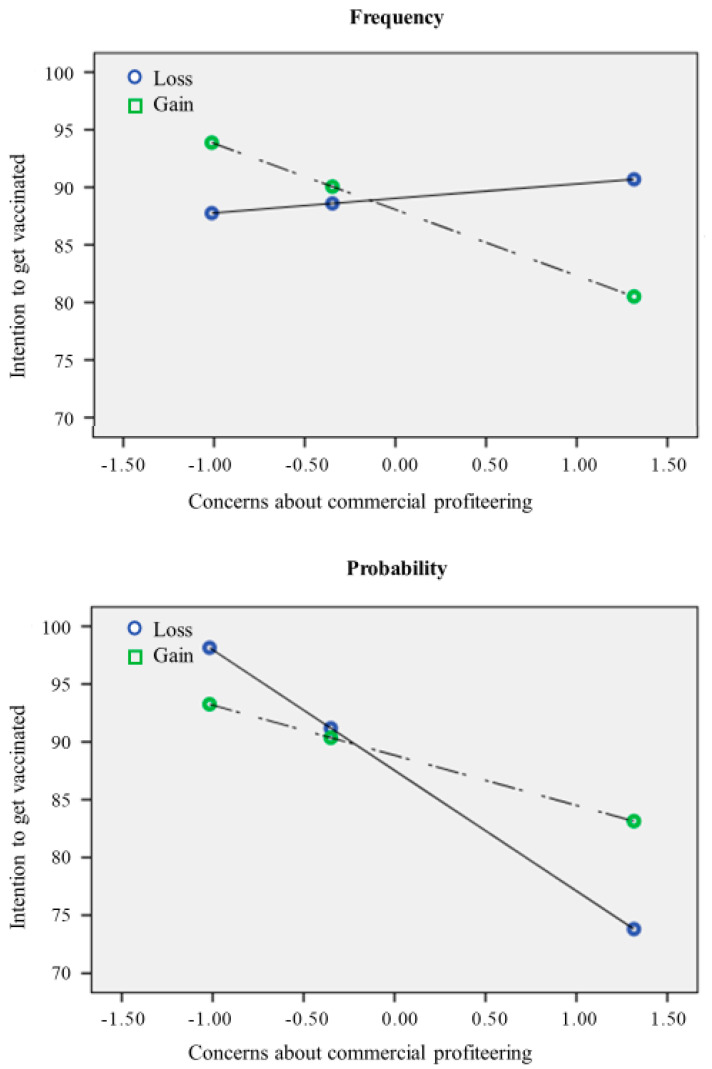
The relationship between concerns about commercial profiteering and intention to get the vaccine.

Concerning the possible effect of framing, the results showed that neither loss-gain nor frequency-probability showed a significant direct effect on intention (respectively: b = 0.14; 95% CI: −2.54, 2.83; b = −0.75; 95% CI: −3.45, 1.95). However, according to our hypotheses, there was a significant interaction between loss-gain and trust in vaccine benefit (b = 3.56; 95% CI: 0.05, 7.08), meaning that the effect of trust in vaccine benefit on intention was stronger in the Gain conditions, rather than in the Loss condition (Figure 2). Moreover, there was a significant interaction between frequency-probability and concerns about commercial profiteering (b = −5.30; 95% CI: −8.98, −1.63), meaning that the negative effect of concerns about commercial profiteering on intention was stronger (more negative) in the probability conditions (Figure 3). The indices of moderated mediation were also consistent with these results, showing that the index of moderated mediation from trust in mainstream information sources through trust in vaccine benefit was significant (b = 0.36; 95% CI: 0.09, 0.63), as well as the moderated mediation index through concerns about commercial profiteering (b = −0.35; 95% CI: −0.64, −0.09). These results thus support our hypotheses about the moderator role of framing in amplifying (or reducing) the effect of the other predictors of intention to get vaccinated.

For the trust in vaccine benefit, we also tested the double interaction (trust in vaccine benefit x loss-gain x frequency-probability), which was significant (b = 13.89; 95% CI: 6.79, 21.00). This means that the moderation effect of loss-gain was higher in the probability condition. Specifically, trust in vaccine benefit on intention had a stronger effect in the Gain conditions (compared to the Loss one), and it was even stronger in the Gain/Probability condition.

Moreover, for the effect of concerns about commercial profiteering, we tested the double interaction (concerns about commercial profiteering × frequency-probability × loss-gain), and this was significant (b = 12.97; 95% CI: 5.65, 20.29). This means there was a moderation effect of frequency-probability, which was higher in the loss condition. In summary, concerns about commercial profiteering had a negative effect on intention, which was stronger (more negative) in the probability conditions and even more negative in the Probability/Loss condition.

## 4. Discussion

Individual vaccination choices are crucial for the success of prevention campaigns. The vaccine decisions might be explained by different inner (e.g., risk perception, attitude, trust, etc.) and external (e.g., social and family environment, constraints, time pressure, etc.) determinants and may change over time. Scientific literature has highlighted that trust in mainstream information sources, vaccine attitude, and framing (in which the information is presented) might impact individual health choices. All mentioned variables have a pivotal role in the definition of the intention to get vaccinated [8,9,13,15,20,21,35]. Available studies reported conflicting results about how these mechanisms interact to shape vaccine decisions for COVID-19 [31].

Our study contributes to theory building by extending the existing conceptualization of message framing in the health domain. Results shed light on the moderator role of framing in amplifying (or reducing) the effect of the other predictors of intention to get vaccinated. Consistently, trust in mainstream information sources regulates each component of the individual vaccine attitude (respectively: trust in vaccine benefit; concerns about commercial profiteering; preference for natural immunity; and worries about unforeseen future). Individuals are more prone to get a vaccine if they can recognize its benefits and have lower concerns about commercial profiteering. Furthermore, the trust in mainstream information sources on individuals’ willingness to get the vaccine is mediated by the trust in vaccine benefit and concerns about commercial profiteering. Individuals are more motivated to get the vaccine if they trust the benefits of the vaccine, as well as lower concerns about commercial profiteering.

Further, this relationship is moderated by the framing used to communicate the vaccine’s effectiveness in counteracting the virus. Specifically, results reported a significant interaction between loss-gain framed messages and trust in vaccine benefit. Specifically, in the loss condition, the influence of trust in vaccine benefit on the intention of getting the vaccine was weaker compared to the gain condition. Furthermore, concerns about commercial profiteering had a more negative effect on intention when participants were exposed to a probability-framed message. This trend is coherent with previous studies, which did not find a direct effect of the frame on health choices [22], but mediating or moderating effects [28,29,30].

Compared with previous research, our studies added insight to comprehend the impact of the frame on vaccine decisions and to make more precise the role of trust in mainstream information sources. Indeed, results advised that trust in the vaccine benefit is an affordance to boost vaccine intention only when we use a gain-framed communication. It might increase trust toward health stakeholders and their preventive actions compared to loss-framed communication. Similarly, concerns about commercial profiteering related to the vaccine have a higher negative impact on vaccine intention when we use probability than frequency. This effect might be explained by numbers framed as a frequency having a greater impact than numbers framed as a percentage [25,36].

In conclusion, the impact of the framing on individual mindset depends on the individuals’ beliefs and attitudes that can affect both the trust in the mainstream sources of information about the virus and the intention to get a vaccine. Due to the complex influence of inner and external determinants on individual health choices, future studies might include other psychocognitive variables, such as health literacy and reasoning strategies. Indeed, it has been proved that people with low health literacy and medical knowledge tend to overestimate their knowledge and mistrust vaccines [37]. Similarly, cognitive fallacies in reasoning may drive toward unhealthy choices [38]. Future vaccination campaigns might try to address the concerns about economic profits from the vaccination campaigns and prefer the use of frequency-framed information compared to probability ones. In addition, loss framing should be avoided when targeting a population with a fair level of trust in the vaccination benefits [31,39]. Furthermore, tailored vaccination campaigns and attention to citizens’ worries and health expectations might help in preventing health inequities [40,41].

### Limitations and Future Studies

The study presents some limitations that constrain the generalizability of our results. Firstly, it has employed a cross-sectional design, so it is not feasible to conclude the causality between variables. Secondly, it is important to take into account that the effect size of the framing effect depends on additional individuals (e.g., thinking style, risk profile, emotional activation) and environmental factors (e.g., time constraints) that are not considered in the study [42]. Furthermore, we enrolled healthy subjects with a high educational level from the general population that are strongly motivated to get the vaccine for COVID-19. A future corrective action might be the creation of clusters of participants with different levels of motivation to get a vaccine and controlled for age and education. Thirdly, some of the measures (like the trust in mainstream sources of information) were ad-hoc and the wording of the description in each framing condition (gain vs. loss—probability vs. frequency) might affect the inconsistent effect of the framing observed when we have compared the four conditions.

The evidence showed that framing studies in within-subjects is more likely to find differences than between-subjects studies. This risk is well-acknowledged in the literature on the framing effect [43,44]. Lastly, our results are based on the perception of gains and losses relying on information and statistics on the general population that have no individual or personal link with the participants.

It is possible that framing information with a more personal style (e.g., speaking directly to the individual and his/her health) might lead to a different perception of risk. It has also been shown that risk information tailored to individual features (i.e., personal behaviours) can increase the perception of risk compared to generic information [45]. Future studies might address this point by comparing information explicitly referring to one’s health (e.g., ‘your risk of…’) with information reporting general statistics. Notwithstanding, we argue that the results obtained in the current study might help healthcare professionals and other stakeholders involved in vaccine campaigns to develop tailored communication strategies according to the target population’s individual psychological, cognitive and behavioral features.

## Figures and Tables

**Table 1:** The two dimensions (loss-gain, and frequency-probability) and how the sample was randomized across the different conditions.

		Loss-Gain	
		0 Loss	1 Gain
Frequency- Probability	0 Frequency	N = 161	N = 156
	1 Probability	N = 158	N = 159

**Table 2:** Means, standard deviations, and the correlations between the key variables under study.

	Mean	SD ^1^	1.	2.	3.	4.	5.	6.	7.
1. Age (18–99)	39.59	16.12	-						
2. Health status (1–5)	3.43	0.79	−0.4180 *	-					
3. Trust in mainstream information sources (0–100)	58.38	15.98	−0.1660 *	0.103	-				
4. Trust of vaccine benefit (1–6)	4.97	0.98	−0.103	0.1890 *	0.443 *	-			
5. Worries about unforeseen future effects (1–6)	3.27	1.20	0.198 *	−0.115	−0.189 *	−0.373 *	-		
6. Concerns about commercial profiteering (1–6)	2.02	109	0.186 *	−0.211 *	−0.433 *	−0.549 *	0.498 *	-	
7. Preference for natural immunity (1–6)	1.94	1.07	0.148 *	−0.133	−0.326 *	−0.445 *	0.452 *	0.643 *	-
8. Intention to get vaccinated (0–100)	88.37	23.24	−0.128	0.159 *	0.353 *	0.624 *	−0.340 *	−0.525 *	−0.405 *

^1^ SD: Standard Deviation; * *p* < 0.001.

## Data Availability

Data are available from the corresponding author upon reasonable request.
